# Clinical Diagnosis of Tuberculosis With Atypical Manifestations Involving the Liver and Spleen With Spontaneous Subscapular Hematoma in a Resource-Limited Setting: A Case Report and Review of Literature

**DOI:** 10.1155/crdi/6629614

**Published:** 2025-11-29

**Authors:** Addisu Assfaw Ayen, Abere Genetu, Bekalu Mekonen Belay, Mengistu Melak Fekadie, Belayneh Dessie Kassa

**Affiliations:** ^1^Department of Internal Medicine, Debre Tabor University, Debre Tabor, Ethiopia; ^2^Department of Internal Medicine, Bahir Dar University, Bahir Dar, Ethiopia; ^3^Department of Adult Health Nursing, Debre Tabor University, Debre Tabor, Ethiopia; ^4^Department of Pediatrics and Child Health Nursing, Debre Tabor University, Debre Tabor, Ethiopia; ^5^Department of Emergency and Critical Care Medicine, Debre Tabor University, Debre Tabor, Ethiopia

**Keywords:** case report, clinical diagnosis, Ethiopia, hepatosplenic tuberculosis, subcapsular hematoma

## Abstract

**Background:**

Isolated liver and spleen involvement of tuberculosis (TB) is a rare presentation.

**Case Presentation:**

A 60-year-old male patient from Bahir Dar City in the Amhara Region of Ethiopia presented with a 4-month history of low-grade fever, significant weight loss, drenching night sweats, poor appetite, exertional shortness of breath, and easy fatigability. Two weeks before his presentation, all symptoms worsened, and the patient started to experience left upper quadrant (LUQ) abdominal pain with a dragging sensation. Abdominal ultrasound showed hepatosplenomegaly with a linear hypoechoic area seen at the upper pole of the spleen, likely infarctions, and periportal lymphadenomegaly was visible. An abdominal CT scan showed a subcapsular hematoma on the upper pole of the spleen. After carefully ruling out both infectious and noninfectious differential diagnoses, we considered extrapulmonary tuberculosis and decided to initiate an antituberculosis trial. Extrapulmonary TB (hepatosplenic TB) was considered, and anti-TB medications were started. On his 6-month follow-up and evaluation, his symptoms were improved with normal physical findings and normal investigation findings.

**Conclusion:**

Even though isolated hepatosplenic TB is a rare condition, it has to be considered especially in low socioeconomic communities after excluding other alternative diagnoses.

## 1. Introduction

Tuberculosis (TB) is one of the major infectious health problems across the world, with a significant impact on low socioeconomic countries like Ethiopia [1]. According to the 2021 World Health Organization report, almost 10 million people worldwide were affected by TB, among which 1.5 million deaths per year were accounted for by TB [2]. TB may present with a variety of manifestations that usually involve the lungs; however, in sporadic cases, extrapulmonary manifestations may develop as well, especially in patients with acquired or congenital immunodeficiency [1]. Hematogenous spread may occur through both the hepatic artery and the portal vein [3]. Hepatosplenic involvement is a rare manifestation of TB, and isolated involvement with pulmonary involvement is even rarer [4]. In this case report, we present a rare case of isolated hepatosplenic TB with spontaneous splenic hematoma. This study was previously made available as a preprint on Research Square to facilitate early dissemination of findings. The current submission has not been altered since the preprint publication [5].

## 2. Case Presentation

A 60-year-old male patient from Bahir Dar in the Amhara Region of Ethiopia presented with low-grade fever, significant weight loss, drenching night sweats, poor appetite, exertional shortness of breath, and easy fatigability for 4 months in March 2022. The patient also experienced vertigo, blurring of vision, and tinnitus for 2 months. Two weeks before his hospital visit, all the above symptoms worsened, and he also started to experience left upper quadrant (LUQ) abdominal pain with a dragging sensation. Otherwise, he had no history of cough, orthopnea, body swelling, bleeding from any site, or swelling around the neck, axilla, or groin region. The patient admittedly drinks alcohol regularly on a daily basis including beer. The patient has no medical history of diabetes, hypertension, or any other chronic illness. For the above complaints, he repeatedly visited a private hospital nearby and was repeatedly treated with unspecified oral and intravenous medications, but there was no improvement.

During the initial evaluation, acute-on-chronic disease manifestation was observed, with a pulse rate of 100 to 108 per minute, a temperature of 37.9°C to 38.5°C, a respiratory rate of 20 breaths per minute, and normal blood pressure and oxygen saturation. Other pertinent positive findings were pale conjunctiva, the spleen palpable 8 cm along the splenic growth line, and the liver palpable 4 cm below the right costal margin with a total liver span of 18 cm, normal chest findings, and no peripheral lymphadenopathy.

**Figure 1:**
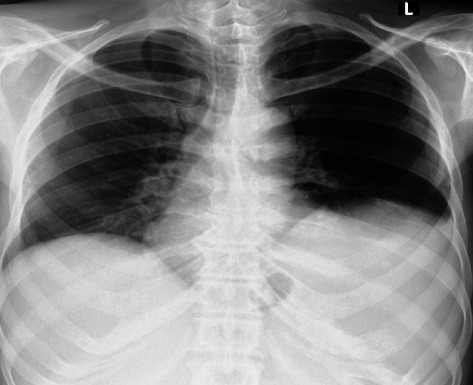
Normal PA chest X-ray with an elevated left hemidiaphragm.

On investigation, the complete blood count showed a WBC count of 2.9 × 10^3^ with neutrophils 51%, lymphocytes 35%, Hgb/Hct 6.9 g/dL/19.6%, MCV 76.7 fL, MCH 27.9, RDW-CV 19.6, and platelets 38 × 10^3^. The erythrocyte sedimentation rate (ESR) was 90 mm per hour. HIV screening, hepatitis B surface antigen, hepatitis C virus antibody, and rk39 were all negative. Liver enzymes, liver function tests, renal function tests, fasting blood glucose, serum electrolytes, peripheral morphology, and bone marrow aspiration were normal. Echocardiography reflected minor mitral calcification. Chest X-ray (Figure 1) showed an elevated left hemidiaphragm with normal parenchyma. Abdominal ultrasound showed nonspecific hepatomegaly (20 cm) and splenomegaly (21 cm) with a linear hypoechoic area seen at the upper pole of the spleen, likely infarctions, and also a few small periportal lymphadenopathies.

**Figure 2:**
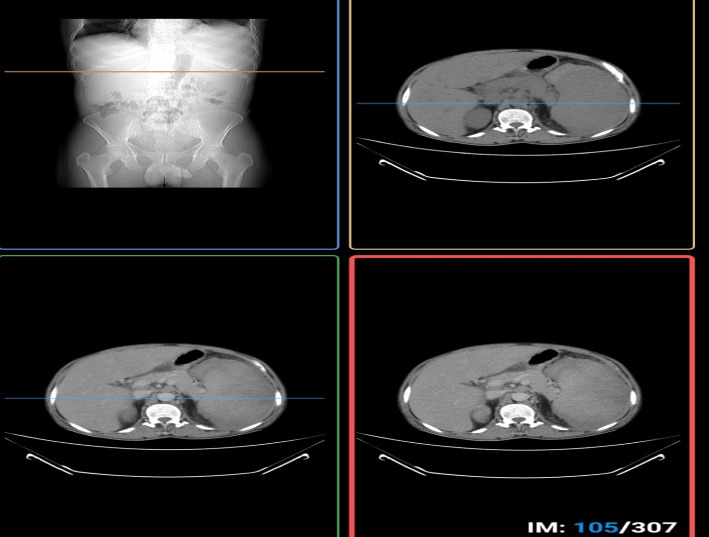
Abdominopelvic CT showing hepatomegaly and splenomegaly with subcapsular hematoma.

Abdominopelvic CT scan (Figure 2) showed that the liver was 20.4 cm in size, and the parenchyma had homogeneous intermediate attenuation or mass. In a postcontrast study, there was no abnormal parenchymal enhancement. The spleen was 22 cm in size, with homogeneous intermediate soft tissue density, and there was a 4.6-cm splenic laceration in the upper third of the spleen associated with 13.4 by 3.4 cm measuring intermediate density (about 59 HU) focus on the anterior–medial aspect of the spleen which was subcapsular hematoma. There were also discrete, mildly enlarged preaortic lymph nodes, the largest measuring 1.7 cm in size.

After a thorough evaluation, extrapulmonary TB was considered. After carefully ruling out both infectious and noninfectious differential diagnoses, we considered extrapulmonary TB and decided to initiate an anti-TB trial, and then, anti-TB medications were initiated with 2RHZE/4RH and pyridoxine. Then, at his first-month follow-up after anti-TB initiation, the patient had no fever, abdominal pain, night sweats, or cough. His easy fatigability decreased, and the patient started to gain weight with an improved appetite. On investigation, the complete blood count was normal, except that the hemoglobin level was 9.5 g/dL, and abdominal ultrasound showed hepatomegaly (17.2 cm but decreased in size from the previous) with a smooth contour and splenomegaly (18 cm but decreased in size from the initial size) with an upper-pole subcapsular hypoechoic collection. Then, anti-TB was continued, and at the second-month follow-up, all his symptoms improved; investigation showed a normal complete blood count, and an abdominal ultrasound showed a liver of normal size with homogeneous echopattern and smooth contour. No focal lesion was seen. The spleen was enlarged (16.5 cm) and had a homogeneous echopattern, and no focal lesion was seen. After that, we continued anti-TB with the 4RH regimen, and he was provided an appointment for a follow-up visit at the end of 6 months of treatment. At the 6-month evaluation, at the end of September 2022, all his symptoms improved with normal physical findings, and all the investigations were normal. We declared a cure and discharged him from follow-up.

## 3. Discussion

Isolated liver and spleen involvement of TB is a rare presentation of TB because mycobacteria need oxygen for survival and development, which is lacking in these organs [6]. TB mostly affects immunosuppressed individuals such as malnourished as our patient was emaciated; alcoholic individuals, as our patient frequently consumed local alcohol; and people of low socioeconomic status, as Ethiopia is a low-socioeconomic country and our patient lives in a rural area with a poor socioeconomic level. Other risk factors for TB include prisoners, children under 5 years of age, the elderly, those living in TB-endemic areas, and healthcare workers [3].

The most commonly involved extrapulmonary sites for TB include lymph nodes, followed by pleura, genitourinary system, musculoskeletal system, gastrointestinal system, and neurologic involvement [1]. Most patients who presented with extrapulmonary manifestations of TB have concomitant pulmonary involvement and rarely present with isolated organ involvement such as our patient who had isolated hepatosplenic involvement; most patients with hepatosplenic TB present with nonspecific manifestations like fever, weight loss, easy fatigability, abdominal pain, and organomegaly but can be asymptomatic and diagnosed incidentally [7]; our patient had constitutional symptoms with organomegaly.

The diagnosis of isolated hepatosplenic TB is not straightforward, as it needs a high index of suspicion in those patients who present with nonspecific manifestations, and always consider it as a differential diagnosis in those patients who have multiple hypodense lesions in the liver and spleen, especially in TB-endemic areas [8]. In addition to basic investigations, hepatosplenic TB diagnosis needs radiological and pathological examinations in which radiologic findings can be an abscess, macronodular, and micronodular among which micronodular is the most common and abscess is a sporadic finding [9]. On imaging findings, most hepatosplenic TB will have abscess and rupture, but our patient had a splenic hematoma [10]. Histopathologic findings of granulomas surrounded by epithelioid cells, lymphocytes, and Langhans giant cells with central caseous necrosis from a biopsy or fine-needle aspiration from lesions will be helpful for the diagnosis of hepatosplenic TB even though there will be a risk of rupture; however, we did not perform tissue sampling in our patient due to a resource-limited setting [11]. Ziehl–Neelsen staining or Mycobacterium PCR could also be performed on histopathologic samples to confirm the diagnosis [12]. However, these tests were not conducted in this case due to the patient's condition and resource limitations.

The mainstay of hepatosplenic TB treatment is antitubercular therapy with 2 months of isoniazid, rifampin, ethambutol, and pyrazinamide (2RHZE) followed by 4 months of isoniazid and rifampin (4RH) plus pyridoxine, and mostly they will respond well to chemotherapy alone as our patient did with chemotherapy alone [12], but rarely patients may require surgical treatment when it ruptures and complications arise [13].

## 4. Conclusion

TB is a common disease but has variable atypical manifestations, as our patient had a splenic hematoma and rare organ involvement, from which isolated hepatosplenic TB is a rare condition with nonspecific manifestations and radiologic findings. Even though it is difficult to diagnose TB with histopathologic examination in a resource-limited setting, it can be diagnosed with a high index of suspicion and imaging findings after excluding alternative diagnoses.

## Data Availability

The authors have nothing to report.

## Nomenclature

cm: Centimeter
CT: Computed tomography
ESR: Erythrocyte sedimentation rate
HCT: Hematocrit
Hgb: Hemoglobin
HIV: Human immunodeficiency virus
HU: Hounsfield units
LUQ: Left upper quadrant
mm: Millimeter
PA: Posteroanterior
2RHZE: Isoniazid, rifampin, ethambutol, and pyrazinamide for 2 months
4RH: Rifampicin and isoniazid for 4 months
WBC: White blood cell
